# Feasibility of an estimated method using graduated utensils to estimate food portion size in infants aged 4 to 18 months

**DOI:** 10.1371/journal.pone.0197591

**Published:** 2018-06-07

**Authors:** Jennifer Bradley, Sarah West-Sadler, Emma Foster, Jill Sommerville, Rachel Allen, Alison M. Stephen, Ashley J. Adamson

**Affiliations:** 1 Human Nutrition Research Centre, Institute of Health & Society, Newcastle University, Newcastle upon Tyne, United Kingdom; 2 MRC Human Nutrition Research, Elsie Widdowson Laboratories, Cambridge, United Kingdom; 3 Public Health England, London, United Kingdom (formerly the Food Standards Agency); 4 Department of Nutritional Sciences, School of Biosciences and Medicine, Faculty of Health and Medical Sciences, University of Surrey, Guildford, Surrey, United Kingdom; McMaster University, CANADA

## Abstract

The Diet and Nutrition Survey of Infants and Young Children (DNSIYC) was carried out in 2011 to assess the nutrient intakes of 4 to 18 month old infants in the UK. Prior to the main stage of DNSIYC, pilot work was undertaken to determine the impact of using graduated utensils to estimate portion sizes. The aims were to assess whether the provision of graduated utensils altered either the foods given to infants or the amount consumed by comparing estimated intakes to weighed intakes. Parents completed two 4-day food diaries over a two week period; an estimated diary using graduated utensils and a weighed diary. Two estimated diary formats were tested; half the participants completed estimated diaries in which they recorded the amount of food/drink served and the amount left over, and the other half recorded the amount of food/drink consumed only. Median daily food intake for the estimated and the weighed method were similar; 980g and 928g respectively. There was a small (6.6%) but statistically significant difference in energy intake reported by the estimated and the weighed method; 3189kJ and 2978kJ respectively. There were no statistically significant differences between estimated intakes from the served and left over diaries and weighed intakes (p>0.05). Estimated intakes from the amount consumed diaries were significantly different to weighed intakes (food weight (g) p = 0.02; energy (kJ) p = 0.01). There were no differences in intakes of amorphous (foods which take the shape of the container, e.g. pureed foods, porridge) and discrete food items (individual pieces of food e.g. biscuits, rice cakes) between the two methods. The results suggest that the household measures approach to reporting portion size, with the combined use of the graduated utensils, and recording the amount served and the amount left over in the food diaries, may provide a feasible alternative to weighed intakes.

## Introduction

The current National Diet and Nutrition Survey (NDNS) rolling programme studies the intakes of children and adults from the age of 18 months in the UK [1]. However, nutrition in early infancy is also of great importance, influencing health in childhood and adulthood [2–4]. Current UK recommendations state that infants should be exclusively breastfed for the first 6 months of life, around which point solid foods can be introduced [5, 6]. It is therefore valuable to assess compliance with recommendations, such as those for infant feeding, to build a strong evidence base to guide future health policies. To capture the intakes of this younger population, the Diet and Nutrition Survey of Infants and Young Children (DNSIYC) was carried out in 2011 in the UK to assess nutrient intakes of 4 to 18 month old infants on a national scale; full details of the DNSIYC are described elsewhere [7]. The aims of DNSIYC were to provide quantitative information on the food and nutrient intakes, sources of nutrients and nutritional status of a representative sample of young infants. This paper will describe pilot work that was carried out prior to the main stage of DNSIYC, to determine the impact of using graduated utensils to estimate food portion sizes in 4 to 18 month old infants.

The suitability of any dietary assessment method depends upon the study objectives. A weighed intake method is often regarded as the ‘gold standard’ in dietary assessment as it provides an objective quantification of portion sizes and does not rely on memory to the same extent as dietary recall-based methods [8] (there is some reliance on memory in terms of remembering to weigh foods before eating and remembering to record all foods and drinks consumed). However, the time-consuming nature of the method can place a high level of burden on participants, leading to biased food records and reduced participant recruitment rates [9]. Historically NDNS used 7-day weighed intakes for dietary assessment [10, 11]; however due to a fall in survey response rates, a 4-day estimated intake method was chosen for the Rolling Programme to reduce participant burden and optimise response rates [12]. Because of the success of NDNS in achieving satisfactory response [1] and to enable comparisons with NDNS data, a 4-day estimated food diary was the chosen method for DNSIYC.

Previous studies have investigated the accuracy of dietary assessment methods in infants and young children. Lanigan et al (2001) compared estimated intakes using standard household measures with weighed intakes in 6 to 24 month old infants and found no significant differences for mean energy intakes and mean intakes of energy-yielding nutrients [13]. However a systematic review of the validity of dietary assessment methods in children by Burrows et al (2010), found that weighed food records provided the best estimate for younger children aged 0.5 to 4 years [14].

It is difficult to record accurate food intakes in this age group as much of the food offered is wasted, often ending up on the floor or soaked into clothes. Due to the nature of the foods eaten by young infants, which tend to be pureed or soft foods, it may be easier for parents to measure and record the volume using graduated utensils such as measuring spoons and containers. To our knowledge, there are no studies which have investigated the use of such utensils to aid portion size estimation. The objectives of the pilot study were to assess whether the provision of graduated utensils altered either the foods given to infants or the amount consumed by comparing estimated intakes to weighed intakes. The overall aim was to determine the feasibility of the method for the main stage of DNSIYC. Four-day estimated intakes using the utensils were compared with 4-day weighed intakes. (Preliminary results from this study have been published previously [15]).

## Materials and methods

### Subjects

Posters advertising the study were displayed in local community centres and parent and baby groups, and recruitment fliers were distributed by researchers in Newcastle city centre to parents/guardians with young infants. The target was to recruit 50 participants. Eligible participants were parents of 4 to 18 month old infants living in Newcastle upon Tyne or the surrounding area.

The study was conducted according to the guidelines laid down in the Declaration of Helsinki and all procedures involving human subjects were approved by the Newcastle University Ethics Committee. Written informed consent was obtained from all parents for their child’s participation.

### Study design

Parents/guardians were asked to complete two 4-day food diaries; one a 4-day weighed intake (WI) using weighing scales provided, and a second 4-day estimated intake (EI) using the graduated utensils provided. The order of administration was randomised. Prior to commencing the study, parents were visited at home and an explanation of how to complete the food diary and use the equipment was given. Parents were asked to complete both food diaries over a 2 week period.

The general layout of the 4-day food diary was the same for both EI and WI [16]. Information on the time of consumption, where and who the infant was with, the food and/or drink consumed including brand names and the amount of food served and the amount left over (or the amount consumed only, see section on estimated method) was collected. Parents were asked to collect wrappers and labels where appropriate. For home-cooked foods, there was space in the diary to write recipes, including ingredients and cooking methods. For breastfed infants, parents were asked to record the duration of feeding in minutes or, if expressed, to write the volume given.

Contact details for the Research Assistant were given to the parent, should they have had any problems or queries while they were taking part. Once the study period was over, a follow-up interview took place to check the food diary for missing information and to make additional notes such as cooking methods used. Parents were then given the equipment for study period two and the procedure followed as above. Once the parent had completed both the weighed and the estimated diaries, we asked which recording method they preferred and why. Responses were written on a standardised form, no voice recordings were taken during the interviews, and no formal analysis was conducted. The information was collected purely to see if there was an overall preference on recording method.

### Estimated method

The utensils provided included a set of six Tala™ measuring spoons (1.25ml-15ml), four Beaba™ graduated storage pots (2x150ml and 2x300ml) and a Vital Baby^®^ 3-stage trainer cup.

Parents were informed that the graduated pots were useful for measuring amorphous foods, for example pureed foods, porridge and yoghurt. The measuring spoons were useful for scraping out leftovers from the infant’s bowl or from the graduated pot and measuring the amount. For discrete food items parents could state the number of items consumed, and describe the size, for example 1 thick slice of white bread, 3 baby rice cakes, ½ medium banana. Parents were advised that if the infant already used a drinking cup with graduations, they could continue to use this to measure liquids. However a drinking cup was provided with the equipment.

For EI, two diary formats were tested; parents were randomly assigned to one format. Half of the parents completed estimated diaries in which they recorded the amount of food served and the amount left over (this required processing by coders to calculate the amount consumed), and the other half recorded the amount of food consumed only (if there was any food left over, this required the parent to calculate the amount consumed). EI from both diary types were compared to WI to assess the relative accuracy against the more established WI method.

### Weighed method

For WI, a demonstration of how to use the weighing scales (accurate to 1g) was given. As with EI, liquids were measured using the graded side on the infant’s cup or bottle. One diary format was used for WI.

### Conversion of volume to weight

Volume and household measurements in the estimated diaries were converted to weights using conversion factors. Standard protocols from MRC Human Nutrition Research (HNR) Elsie Widdowson Laboratories in Cambridge were followed by researchers at Newcastle University to obtain food densities for all foods consumed in the estimated diaries. An average of five measurements was calculated for weight of food per 100ml; weight per household teaspoon and tablespoon; weight per study teaspoon and tablespoon (household spoons were basic stainless steel spoons, and study spoons were the spoons included in the equipment provided). For spoons, an average of a heaped and level spoonful was calculated. All food weights were sent to MRC HNR, where conversion factors were calculated.

The weighed and estimated diaries were also sent to MRC HNR for coding. Food diaries were coded using the Government’s NDNS Nutrient Databank. Breast milk intake was calculated based on 13.5g/minute with a maximum intake of 135g per feed for infants aged 4 to 7 months, and 10g/minute with a maximum of 100g per feed for those aged 8 to 18 months. A maximum of 1200g/day of breast milk per infant was applied. This was the method adopted by MRC HNR for DNSIYC and is based on work by Mills and Tyler (1992) [7, 17].

### Statistical methods

Statistical analyses were conducted using IBM SPSS for Windows, version 19 (Armonk, NY: IBM Corp). The following were tested for statistical significance:

- difference between WI and EI for reported food weight consumed (g) and reported energy intake (kJ);
- difference between WI and EI using amount consumed diaries for reported food weight consumed (g) and reported energy intakes (kJ)
- difference between WI and EI using served & left over diaries for reported food weight consumed (g) and reported energy intakes (kJ)
- difference between WI and EI for reported portion size (g) and reported energy intake (kJ) of amorphous foods and discrete food items

Wilcoxon signed rank test were conducted as the data were not normally distributed. Median and IQR are reported.

## Results

### Study sample

Fifty participants were recruited, however one participant had to withdraw during the study due to illness. In total, the diets of 24 infants aged 4 to 8 months (mean age 6.4 months) and 25 infants aged 9 to 18 months (mean age 13.0 months) were obtained. Eighty percent of participants completed both diaries over a two week period; 20% completed over three weeks.

### Average daily intakes

Table 1 shows the median daily intakes of food weight (g) and energy (kJ) for the two methods (WI and EI) and for the two diary formats (amount consumed and served & left over). On average the weight of food reported was 2.5% higher in the estimated diary compared with the weighed diary and reported energy intake was 8.1% higher. Although average daily EI were higher than WI, there were no statistically significant differences between weighed and estimated food weight. There was a statistically significant difference between weighed and estimated energy intake (p = 0.02).

**Table 1:** Comparison of median daily intakes of food weight (g) and energy (kJ) for the weighed and estimated methods and for the two estimated diary formats; amount consumed and served & left over.

Measures	Weighed intake (n = 49^a^)		Estimated intake								
			Average of all estimated intake diaries^b^ (n = 49)			Amount consumed (n = 25)			Served & left over (n = 24)		
	Median	IQR	Median	IQR	Difference between weighed and estimated methods (p-value)	Median	IQR	Difference between weighed and amount consumed method (p-value)	Median	IQR	Difference between weighed and served & left over method (p-value)
Food weight (g)	928	215	980	258	0.09	1035	260	0.02	965	249	1.00
Energy (kJ)	2978	780	3189	938	0.02	3309	1062	0.01	3167	572	0.57

^a^One participant withdrew from the study due to illness

^b^This is the average of all the estimated food diaries (amount consumed and served & left over formats); IQR: Interquartile range

Forty-five percent of the sample had mean daily estimated food intakes (g) which were within 10% of the weighed food intakes. Ninety-six percent were within 50% of weighed food intakes. Infants in this age group are growing quickly, and food intakes may increase on a weekly basis, however we did not see a pattern of intakes increasing in the second assessment.

### Method of reporting portion size consumed in the estimated diary

Median weight of food consumed and energy intakes were higher for diaries where the amount consumed was recorded compared to when served and leftovers were recorded (Table 1). EI were closer to WI in the diaries where the amount served & left over were recorded. There were no statistically significant differences between EI using amount served & left over and WI. However, there was a significant difference between EI using amount consumed and WI for weight of food (p = 0.02) and energy (p = 0.01).

**Fig 1:**
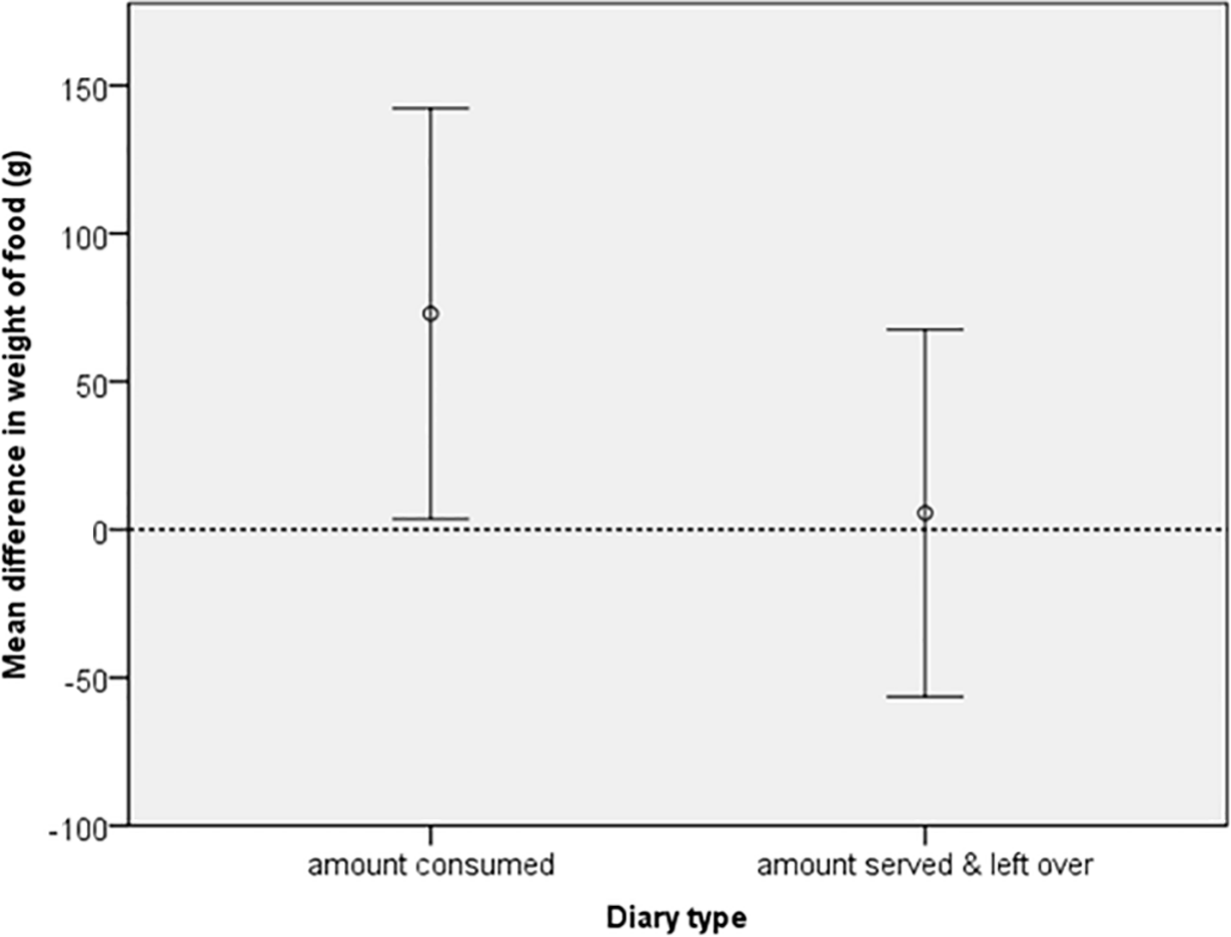
Error bars (95% CI) of mean difference between estimated and weighed food weight (g). Two estimated diary formats were tested; one in which the parent recorded the amount of food consumed and a second where the parent recorded the amount of food served and left over.

**Fig 2:**
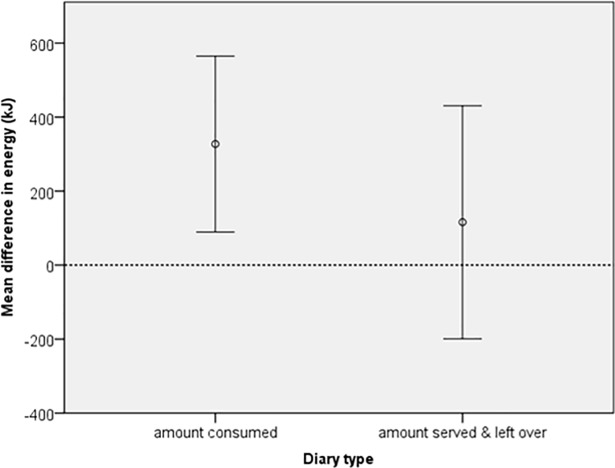
Error bars (95% CI) of mean difference between estimated and weighed energy (kJ). Two estimated diary formats were tested; one in which the parent recorded the amount of food consumed and a second where the parent recorded the amount of food served and left over.

The mean difference between EI and WI for both diary formats for food weight (g) and energy (kJ) can be seen in Figs 1 and 2. A mean close to zero (indicated by the dotted line) would suggest that there is little difference between EI and WI. The figures show that EI from the served and left over diaries were closer to WI compared to amount consumed diaries, which on average gave higher intake values.

### Amorphous and discrete food types

For the estimated method, amorphous foods were likely to have been measured using the graduated pots and spoons, and for discrete food items, household measures were used. To determine whether there were any differences in portion sizes between the two methods, the 50 most commonly consumed foods were assigned a ‘food-type’ based on its properties. The two food types of interest were amorphous and discrete food items. Intakes for the two methods for both food types were comparable (Table 2). No statistically significant differences were found between WI and EI for the portion size of amorphous foods or discrete food items.

**Table 2:** Median portion size (g) and energy (kJ) for amorphous foods and discrete food items for weighed and estimated method (*n =* number of occasions each food type consumed).

		Method			
		4–8 month age group		9–18 month age group	
Food type		Weighed	Estimated	Weighed	Estimated
**Amorphous**	n	52	61	80	96
Food weight (g)	Median [IQR]	44 [25]	46 [23]	45 [33]	55 [22]
Energy (kJ)	Median [IQR]	113 [151]	146 [158]	211 [133]	183 [99]
**Discrete**	n	164	119	231	203
Food weight (g)	Median [IQR]	19 [18]	21 [12]	23 [13]	21 [20]
Energy (kJ)	Median [IQR]	107 [70]	115 [111]	147 [144]	168 [172]

## Discussion

The main objectives of the study were to determine the extent of the difference between estimated intakes using measuring equipment and weighed intakes in 4 to 18 month infants, in terms of the amount and types of foods consumed.

The results indicated that EI supported by the use of graduated utensils and reported as served and left over amounts in the food diaries, provided very similar results to WI, and was a feasible method which could be adopted for the main stage of DNSIYC. However, discussions with parents during the final interview found that the majority preferred the weighed method (66%), and although they found it more time-consuming to weigh every food rather than estimate, they preferred to record the measurements accurately. However, this was the preference of a relatively small sample of parents (n = 49), who volunteered themselves for participation. In the main stage of DNSIYC, participants are first identified and then invited to participate, quite a different scenario to volunteering and hence to ensure a representative sample was obtained, a less burdensome estimated method was ultimately the chosen method for DNSIYC.

For the purposes of the pilot study, we considered WI to be the ‘gold standard’ method. With this in mind, intakes recorded using the amount served and left over diaries were closer to WI compared to intakes recorded using the amount consumed diaries. It was recommended that asking parents to record the amount served and the amount left over was the best option for reporting portion size in the main stage of DNSIYC. Children often leave a proportion of the foods which they are served, and therefore, in some cases, parents may find difficulties estimating the portion of food consumed as they are being asked to conceptualise an amount of food they had never actually seen [18].

There were no differences in the intakes of amorphous and discrete food items by method, suggesting that the household measures approach to reporting portion size, with the combined use of the graduated utensils would provide a feasible alternative to weighing, and would not influence the amounts given to the infant.

However, it is important to acknowledge that the study sample was small; the numbers were recruited based on assessing the feasibility of the method and not the accuracy. Each parent completed a weighed and an estimated food diary, however these covered different days and therefore many factors may have influenced the amount and the types of foods given to the child. As the aim was to complete both assessments within a two week period, it was not always possible to cover the same days, as researchers worked around parents’ other commitments. In addition, the completion of the first assessment may have influenced the way the parent completed the second assessment. In order to reduce the potential effects of this, the order of assessments was randomised.

Before the main stage of DNSIYC commenced, a dress rehearsal phase was conducted with 188 participants, which implemented the estimated method using graduated utensils. A review of the coding rates during the dress rehearsal found the use of the graduated implements resulted in a low coding rate (number of diaries coded per week) and a high number of queries (problems that could not be resolved by the coder alone and needed discussion with others and/or decision by more highly qualified members of the dietary assessment team) [7]. Based on the expected number of participants in the main stage of DNSIYC, over 1800, there was concern that this would have major resourcing consequences for the main survey. The final decision was therefore to proceed with a household measures estimated approach for dietary data collection, without the use of graduated utensils. For the main stage, parents were asked to record the amount served and the amount left over.

This research has demonstrated that an estimated intakes method combining the use of graduated containers with household measures is a practical and feasible alternative to weighed intakes in this age group. The use of estimated diaries in which the parent records the amount served and the amount left over, shifts the burden of recording the amount of food consumed from the participant to the researcher. This in turn may result in more accurate estimations however there may be implications for feasibility in research projects conducted at large scale.

Online dietary assessment tools are becoming a popular choice for food and nutrition surveys, as the low-cost nature and standardised process, makes them an attractive option for large scale surveys [19–21]. At the time of DNSIYC, web-based methods were still in their infancy. In order for the data to compliment with the wider NDNS, there was a requirement to keep the methodology as consistent as possible with the NDNS methodology. In future it is likely that online dietary assessment methodologies will become integrated into national dietary surveys such as NDNS [12].

## Supporting information

S1 Table Energy and weight of food reported in weighed and estimated weight food diaries. (XLSX)
